# Supplementary material for: Identification of disease treatment mechanisms through the multiscale interactome
Source: Nat Commun. 2021 Mar 19;12:1796. doi: 10.1038/s41467-021-21770-8 (PMC7979814; doi:10.1038/s41467-021-21770-8)
Supplement: Supplementary file 1 — Supplementary Information [file 41467_2021_21770_MOESM1_ESM.pdf]

Supplementary materials for

Identification of disease treatment mechanisms through the multiscale interactome

Camilo Ruiz,[1,2] Marinka Zitnik,[3] Jure Leskovec[1,4,*]

[1]Computer Science Department, Stanford University, Stanford, CA 94305, USA

[2]Bioengineering Department, Stanford University, Stanford, CA 94305, USA

[3]Biomedical Informatics Department, Harvard University, Boston, MA 02115, USA

[4]Chan Zuckerberg Biohub, San Francisco, CA 94158, USA

[*]Corresponding author. Email: jure@cs.stanford.edu

This PDF file includes:

Supplementary Notes 1 to 5

Supplementary Figures 1 to 18

Other supporting material for this manuscript includes the following. Data and code are available at github.com/snap-stanford/multiscale-interactome.

Supplementary dataset of interactions between drugs and proteins

Supplementary dataset of interactions between diseases and proteins

Supplementary dataset of interactions between proteins and proteins

Supplementary dataset of interactions between proteins and biological functions

Supplementary dataset of hierarchy of interactions between biological functions

Supplementary dataset of approved drug-disease pairs

Supplementary dataset of drug classes according to the Anatomical Therapeutic Chemical Classification

Supplementary dataset of selected gene expression signatures from the Broad Connectivity Map

Supplementary dataset of genetic mutations that alter treatment from PharmGKB

Supplementary dataset of optimized diffusion profiles for drugs and diseases

Supplementary Note 1 Predictive power of diffusion profiles on a molecular-scale interactome.

We find that diffusion profiles improve prediction of what drugs treat a disease, even when computed on a molecular-scale interactome. We compare optimized diffusion profiles on the molecular-scale interactome to a state of the art proximity metric[1,2] (Methods). We find that diffusion profiles improve prediction relative to proximity (AUROC = 0.674 vs. 0.620, +12.3%; Average Precision = 0.078 vs. 0.065, +20.0%; Recall@50 = 0.317 vs. 0.264, +20.0%). All evaluation metrics are reported on a held-out set, averaged across five-fold cross validation (Methods).

Supplementary Note 2 Selecting ranges for optimization of scalar weights

The multiscale interactome utilizes optimized edge weights to propagate the effects of drugs and diseases to proteins and biological functions. To optimize these edge weights, we conduct a hyperparameter sweep and allow each hyperparameter to vary over a prespecified range. In our initial sweep, we conduct coarse explorations for each hyperparameter in the range [1, 10] and then conduct a finer sweep in the range [3, 9]. Notice that in the calculation of a diffusion profile, the relative values of two hyperparameters dictate the likelihood that the random walker visits one node type rather than another. For example, if $w_{\text{biological function}} = 9$ and $w_{\text{protein}} = 3$, then a random walker adjacent to both a biological function node and a protein node is three times more likely to visit the biological function node than the protein node. If instead $w_{\text{biological function}} = 3$ and $w_{\text{protein}} = 1$, then the random walker is still three times more likely to visit the biological function node than the protein node. In other words, the relative values between the weights affect the propagation but the absolute value of each particular weight does not. By setting the range of each hyperparameter to [3, 9], we thus allow each node type to be jumped to as much as 3X more or less often than other node types.

To ensure that our initial hyperparameter sweep was not too narrow, we conduct a broader hyperparameter sweep and test 560 combinations of edge weight hyperparameters sampled linearly in the range [1, 100] and $\alpha \in [0.667, 1.0]$. Here, each node type can be jumped to as much as 100X more or less often than other node types depending on the sampled weights. The optimal model identified by this expanded hyperparameter sweep did not outperform the model previously described (Methods: Model selection and optimization of scalar weights) in predicting what drug treats a given disease (AUROC: 0.706 vs 0.705; Average Precision: 0.092 vs 0.091; Recall@50: 0.335 vs 0.347). Therefore, we empirically find that our initial hyperparameter range was appropriate and not too narrow.

Supplementary Note 3 Identifying proteins and biological functions relevant to treatment

The multiscale interactome identifies proteins and biological functions relevant to treatment from a large number of possibilities. To explain treatment, the multiscale interactome identifies a subgraph of relevant proteins and biological functions. This subgraph explains how drug-protein, disease-protein, protein-protein, protein-function, and function-function edges compose in a manner that explains treatment. Critically, our case studies describe treatments that cannot be explained by a single protein node, biological function node, protein-protein edge, or protein-function edge. Instead, treatment results from the complex interactions of multiple proteins and biological functions, and a subgraph is necessary to illustrate how these interactions compose to explain treatment. Additionally, note that the multiscale interactome is not meant to identify new protein-function edges or new edges generally. The protein-function edges and other edges in our network are already known. Instead, our model is meant to identify how existing interactions between drugs, diseases, proteins, and biological functions compose to explain treatment.

Moreover, note that the multiscale interactome identifies relevant proteins and biological functions from a large array of possibilities. In each case study, we identify 18 proteins and biological functions relevant to treatment from a network of 17,660 proteins and 9,798 biological functions. Notice that there are 1.22×10^{64} potential choices of proteins and biological functions (27,458 choose 18). (For comparison, note there are only about 7×10^{22} stars in the entire universe.) From this large array of possibilities, diffusion profiles identify a subgraph of proteins and functions that are relevant to treatment. We validate the subgraphs by comparison with externally validated treatment mechanisms. Notably, the knowledge from these externally validated treatment mechanisms is never encoded in our model. Instead, diffusion profiles identify relevant proteins and biological functions from a large array of possibilities. This is the key explanatory benefit of our approach.

Alternative approaches to identify biological functions relevant to treatment. We compared the ability of the multiscale interactome to identify biological functions relevant to treatment with three alternative approaches: selecting GO terms associated with both drug targets and disease proteins, selecting GO terms enriched among both drug targets and disease proteins according to Gene Set Enrichment Analysis[3], and selecting GO terms on shortest paths between drug targets and disease proteins. First, we discuss conceptual limitations of these alternative approaches. Second, we compare these approaches with the multiscale interactome directly for the case studies

presented as Figure 2g and Figure 2h.

Conceptual Issues with Alternative Approaches: Conceptually, these alternative approaches are limited in ways that apply generally regardless of the case study utilized. Selecting common GO terms between drug targets and disease proteins will necessarily prioritize the most popular GO terms that the most proteins connect to (*i.e.*, Biological Process, Cellular Process, Metabolic Process) which are too generic to yield meaningful insight into treatment. Selecting GO terms enriched among drug targets and disease proteins struggles in cases where there are few drug targets or disease proteins. In these cases, few GO terms are enriched and there are few or no common enriched terms, thus yielding little insight. Identifying GO terms that occur on the shortest paths between drug targets and disease proteins fails in cases where the shortest paths between drug targets and disease proteins do not include GO terms. In cases where shortest paths do include the relevant GO terms, diffusion profiles are likely to identify these GO terms anyways since nodes on the shortest paths between drug targets and disease proteins are frequently visited in diffusion profiles. Finally, all three alternative approaches only generate lists of GO terms. By contrast, diffusion profiles identify subgraphs which demonstrate how complex interactions between drugs, diseases, proteins, and biological functions can help explain treatment. Lists of GO terms can at most identify relevant functions but cannot shed light into the drug-protein, disease-protein, protein-protein, protein-function, or function-function interactions that are relevant to treatment. Such complex interactions are important for describing treatment[1,2,4–7].

Example: Treatment of Cryopyrin-Associated Periodic Syndromes by Anakinra. First, consider the treatment of Cryopyrin-Associated Periodic Syndromes by Anakinra (Figure 2h). Cryopyrin-Associated Periodic Syndromes are characterized by immune-mediated inflammation via the Interleukin-1 beta signaling pathway[8]. Anakinra treats Cryopyrin-Associated Periodic Syndromes by binding to a regulator of the Interleukin-1 beta signaling pathway and thus preventing excess inflammation[9,10]. Diffusion profiles capture Anakinra's treatment of Cryopyrin-Associated Periodic Syndromes by affecting immune-mediated inflammation via the Interleukin-1 beta signaling pathway (Supplementary Fig. 14a). Diffusion profiles identify 14 GO terms, 10 of which describe treatment: "inflammatory response," "response to interleukin-1," "IL-1 mediated signaling pathway," "cytokine-mediated signaling pathway," "negative regulation of IL-1 mediated signaling pathway," "regulation of IL-1 mediated signaling pathway," "negative regulation of inflammatory response," "cellular response to IL-1," "cellular response to cytokine stimulus," and "response to cytokine." Diffusion profiles thus capture the key biological functions underlying treatment: regulation of immune-mediated inflammation via the Interleukin-1 beta signaling pathway.

By contrast, none of the alternative approaches capture the key biological function underlying treatment: regulation of immune-mediated inflammation via the Interleukin-1 beta signaling pathway. First if we consider GO terms that are associated with both Anakinra's targets and Cryopyrin-Associated Periodic Syndrome's genes, we find nine such GO terms (Supplementary Fig. 14b). However, these GO terms broadly miss the effect of Anakinra on immune-mediated inflammation and the Interleukin-1 beta signaling pathway. Indeed, eight of these nine GO terms are generic and not useful to describing treatment (*i.e.*, "biological process," "regulation of cellular process," "response to chemical," "biological regulation," "cellular process," "regulation of biological process," "response to organic substance," "response to stimulus"). Second if we consider GO terms enriched among both Anakinra's targets and Cryopyrin Associated Periodic Syndrome's genes, we find that there are no common GSEA GO terms (Supplementary Fig. 14c). Finally if we consider GO terms on shortest paths between Anakinra's targets Cryopyrin Associated Periodic Syndrome's genes, we find that there are no GO terms on the shortest paths between Anakinra and Cryopyrin Associated Periodic Syndromes (Supplementary Fig. 14d). Therefore, the three alternative approaches miss the effect of Anakinra on immune-mediated inflammation and the Interleukin-1 beta signaling pathway. Diffusion profiles thus identify the biological functions related to the treatment of Cryopyrin-Associated Periodic Syndromes by Anakinra more effectively than alternative approaches.

Example: Treatment of Hyperlipoproteinemia Type III by Rosuvastatin. Next, consider the treatment of Hyperlipoproteinemia Type III by Rosuvastatin. Hyperlipoproteinemia is a disease characterized by abnormal levels of cholesterol in the blood[11–15]. Rosuvastatin treats Hyperlipoproteinemia Type III by affecting various biological functions related to cholesterol biosynthesis and metabolism[10,16,17]. Diffusion profiles successfully identify the cholesterol biosynthesis functions relevant to treatment of Hyperlipoproteinemia Type III's by Rosuvastatin (Supplementary Fig. 13a). Diffusion profiles identify 12 GO terms, 5 of which describe cholesterol biosynthesis (*i.e.*, "regulation of cholesterol biosynthetic process," "positive regulation of cholesterol biosynthetic process," "negative regulation of cholesterol biosynthetic process," "cholesterol biosynthetic process," "cholesterol homeostasis"). An additional 3 GO describe triglyceride metabolism, a function dysregulated in Hyperlipoproteinemia Type III[11–15] (*i.e.*, "positive regulation of triglyceride catabolic process," "triglyceride metabolic process," "triglyceride homeostasis"). Diffusion profiles thus capture key biological functions relevant to treatment.

By contrast, two of the three alternative approaches are less effective than diffusion profiles in identifying biological functions that are both specific and relevant to treatment. First, if we consider

GO terms enriched among both Rosuvastatin's target and Hyperlipoproteinemia Type III's genes, we find no common enriched GO terms, thus yielding no insight into treatment (Supplementary Fig. 13c). Second, if we consider GO terms that are associated with both Rosuvastatin's target and Hyperlipoproteinemia Type III's genes, only 1 of 19 identified GO terms is specific and relevant (Supplementary Fig. 13b). The remaining GO terms are generic (*i.e.*, "cellular component organization", "macromolecular complex assembly") and thus not particularly insightful. By contrast, 8 of the 12 GO terms identified by diffusion profiles are specific and relevant. Moreover, the single relevant GO term identified via this alternative approach (*i.e.*, "cholesterol metabolic process") is more generic than the five cholesterol-related GO terms identified via diffusion profiles (*i.e.*, "regulation of cholesterol biosynthetic process," "positive regulation of cholesterol biosynthetic process," "negative regulation of cholesterol biosynthetic process," "cholesterol biosynthetic process," "cholesterol homeostasis"). Indeed in the Gene Ontology, metabolic processes define broad functions that can be altered in many ways. Two specific ways to alter metabolic processes are effects on biosynthesis and biosynthesis regulation[18,19], the functions identified by diffusion profiles. Ultimately, common enriched GO terms and common associated GO terms are less effective than diffusion profiles in identifying biological functions both specific and relevant to treatment.

The third alternative approach, considering GO terms on shortest paths between Rosuvastatin and Hyperlipoproteinemia Type III, performs comparably to diffusion profiles, identifying 4 GO terms related to cholesterol biosynthesis (*i.e.*, "cholesterol metabolic process," "negative regulation of cholesterol biosynthetic process," "cholesterol biosynthetic process," and "cholesterol homeostasis") (Supplementary Fig. 13d). Three of these GO terms overlap with the biological functions identified by diffusion profiles. Here, the comparable performance between diffusion profiles and GO terms on shortest paths makes sense. In cases where relevant biological functions are on shortest paths between drug targets and disease proteins, diffusion profiles will identify these GO terms as they will likely be frequently visited in the drug and disease diffusion profiles. In other treatments, however, the shortest paths between drug targets and disease proteins may not include GO terms. For such treatments, diffusion profiles will provide functional insight not provided by simply identifying GO terms on shortest paths.

Diffusion profiles capture the cholesterol biosynthesis functions relevant to treatment and thus offer an effective approach to identifying the biological functions relevant to treatment.

Supplementary Note 4 The multiscale nature of our network and alternative multiscale interactomes

The multiscale interactome contains both physical interactions between proteins and a hierarchy of biological functions. We consider the network "multiscale" for two reasons. First, biological functions are often defined at the level of molecules (*i.e.*, DNA Demethylation GO:0080111), cells (*i.e.*, Mitotic Cell Cycle GO:0000278), tissues (*i.e.*, Muscle Atrophy GO:0014889), organ systems (*i.e.*, Activation of Innate Immune Response GO:0002218), and the whole organism (*i.e.*, Anatomical Structure Development GO:0048856)[20,21]. Second, the hierarchical relationships between biological functions contains a "detailed implicit ontology of anatomical structures"[22]. This point is made clearer in "GO-Plus"[19] which links biological functions to the cells, tissues, and organs in Uberon[22,23] and the Cell Ontology[24,25] (two anatomical ontologies) that these biological functions affect. For these reasons, we consider the network we constructed multiscale.

We acknowledge that alternative interpretations of "multiscale" may wish that cells, tissues, and organs be explicitly included as nodes in our network along with the anatomical relationships between them. To address this potential interpretation, we construct alternative multiscale interactomes that explicitly represent cells, tissues, organs and the relationships between them (Supplementary Fig. 8, Methods). Specifically, we created three alternative multiscale interactomes that incorporate human-specific subsets of Uberon[22,23], the Cell Ontology[24,25], or both. In these alternative multiscale interactomes, our initial network is expanded such that (1) human cells, tissues, and organs are added as additional nodes, (2) these cells, tissues, and organs have edges between them according to the anatomical hierarchies defined in human subsets of Uberon and the Cell Ontology, and (3) the biological functions in GO are linked to the new cell, tissue, and organ nodes that they affect according to Gene Ontology Plus (GO Plus)[19]. All three of the alternative multiscale interactomes outperform molecular-scale interactomes when predicting what drug treats a disease. The best alternative multiscale interactome (multiscale interactome + Uberon) outperforms a molecular-scale interactome by 15.3% in AUROC (0.715 vs. 0.620), 40.0% in Average Precision (0.091 vs. 0.065), and 29.9% in Recall@50 (0.343 vs. 0.264) (Supplementary Fig. 8). In these alternative multiscale interactomes, our model is still able to effectively predict what drugs treat a disease.

Supplementary Note 5 Variants in network construction and edge weighting

We test whether numerous variants to the edge weighting and network construction approach described in Methods would improve the ability of our model to predict what drugs treats a given disease.

1. The multiscale interactome with all GO biological function terms vs. the multiscale interactome with only biological function terms associated with at least one drug target or disease protein (Supplementary Fig. 9; Methods: Biological function – biological function interactions).

2. The multiscale interactome when all disease-protein edges are treated equivalently (*i.e.*, w_{protein}, w_{disease}) vs. the multiscale interactome when different disease-protein edge types receive different hyperparameter edge weights (*i.e.*, $w_{\text{disease}\rightarrow\text{protein via genomic alteration}}$, $w_{\text{disease}\rightarrow\text{protein via altered expression}}$, $w_{\text{disease}\rightarrow\text{protein via post-translational modification}}$, $w_{\text{protein}\rightarrow\text{disease via genomic alteration}}$, $w_{\text{protein}\rightarrow\text{disease via gene expression}}$, $w_{\text{protein}\rightarrow\text{disease via post-translational modification}}$) (Supplementary Fig. 11).

3. The multiscale interactome when all biological function – biological function edges are treated equivalently (*i.e.*, $w_{\text{biological function}}$, $w_{\text{higher-level biological function}}$, $w_{\text{lower-level biological function}}$) vs. when they are differentiated based on the type of GO edge (*i.e.*, $w_{\text{biological function}}$, $w_{\text{higher-level bf via 'is a'}}$, $w_{\text{lower-level bf via 'is a'}}$, $w_{\text{higher-level bf via 'part of'}}$, $w_{\text{lower-level bf via 'part of'}}$, $w_{\text{higher-level bf via 'regulates'}}$, $w_{\text{lower-level bf via 'regulates'}}$, $w_{\text{higher-level bf via 'positively regulates'}}$, $w_{\text{lower-level bf via 'positively regulates'}}$, $w_{\text{higher-level bf via 'negatively regulates'}}$, $w_{\text{lower-level bf via 'negatively regulates'}}$; "bf" denotes biological function) (Supplementary Fig. 10).

For each variant, we conduct the broad hyperparameter sweep described in Supplementary Note 2. Hyperparameters not mentioned above are defined as in the original model (Methods). Ultimately, we use these variants to predict what drug treats a disease according to the approach previously described (Methods). All optimal models used the correlation distance to compare the drug and disease diffusion profiles. The described variants did not substantially outperform the original model in predicting what drugs treat a given disease (Supplementary Fig. 9, Supplementary Fig. 10, Supplementary Fig. 11).

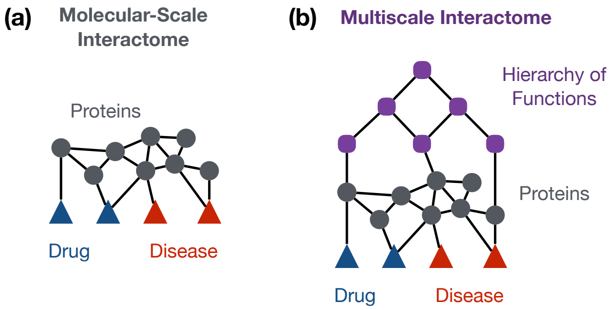

Supplementary Figure 1: The multiscale interactome incorporates both physical interactions between proteins and a hierarchy of biological functions. **(a)** Molecular scale interactomes model drug-disease treatment by primarily considering physical interactions (*i.e.*, drug–protein, disease–protein, and protein–protein edges). **(b)** The multiscale interactome models drug-disease treatment by considering both physical interactions (*i.e.*, drug–protein, disease–protein, protein–protein edges) and a hierarchy of biological functions (*i.e.*, protein–biological function, biological function – higher-level biological function, and biological function – lower-level biological function edges). By modeling both physical interactions between proteins and a hierarchy of biological functions, the multiscale interactome integrates physical and functional relationships in the modeling of drug-disease treatment. The detailed construction of both networks is provided in Methods.

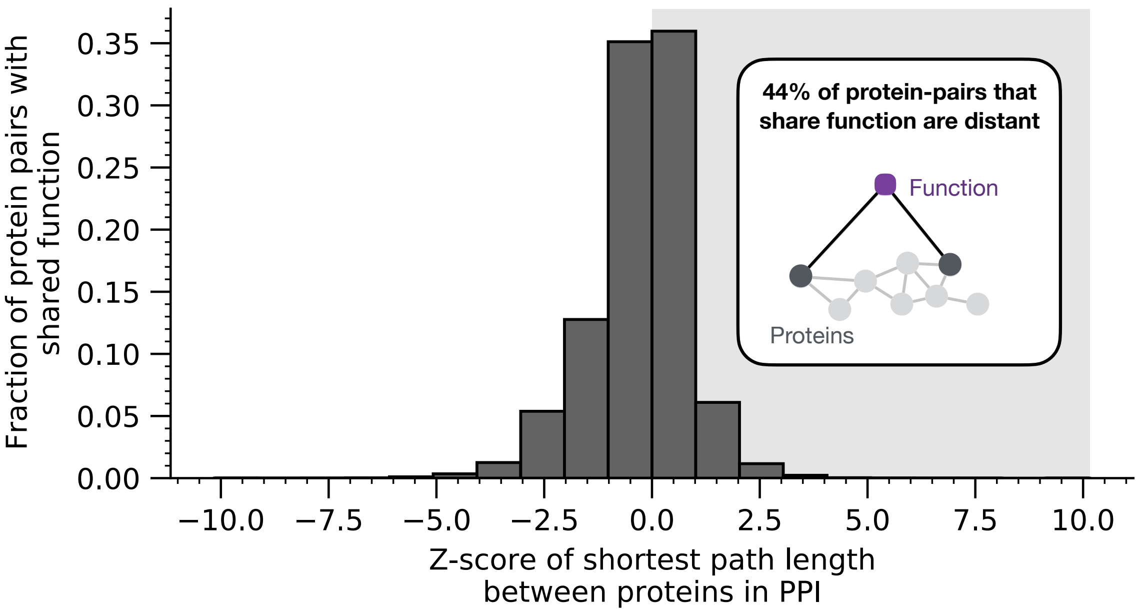

Supplementary Figure 2: Biological functions capture critical relationships between proteins that physical PPI networks alone do not capture. 44% of protein-protein pairs that affect the same biological function are more distant in protein-protein interaction (PPI) network than is expected by random chance. Therefore, proteins may be physically distant yet affect the same function, and biological functions capture critical relationships between proteins that physical protein-protein interaction networks alone do not capture. The shortest path length between each protein-pair in the PPI network that shares a function was compared to a reference distribution of shortest path lengths between protein pairs of similar degree (Methods).

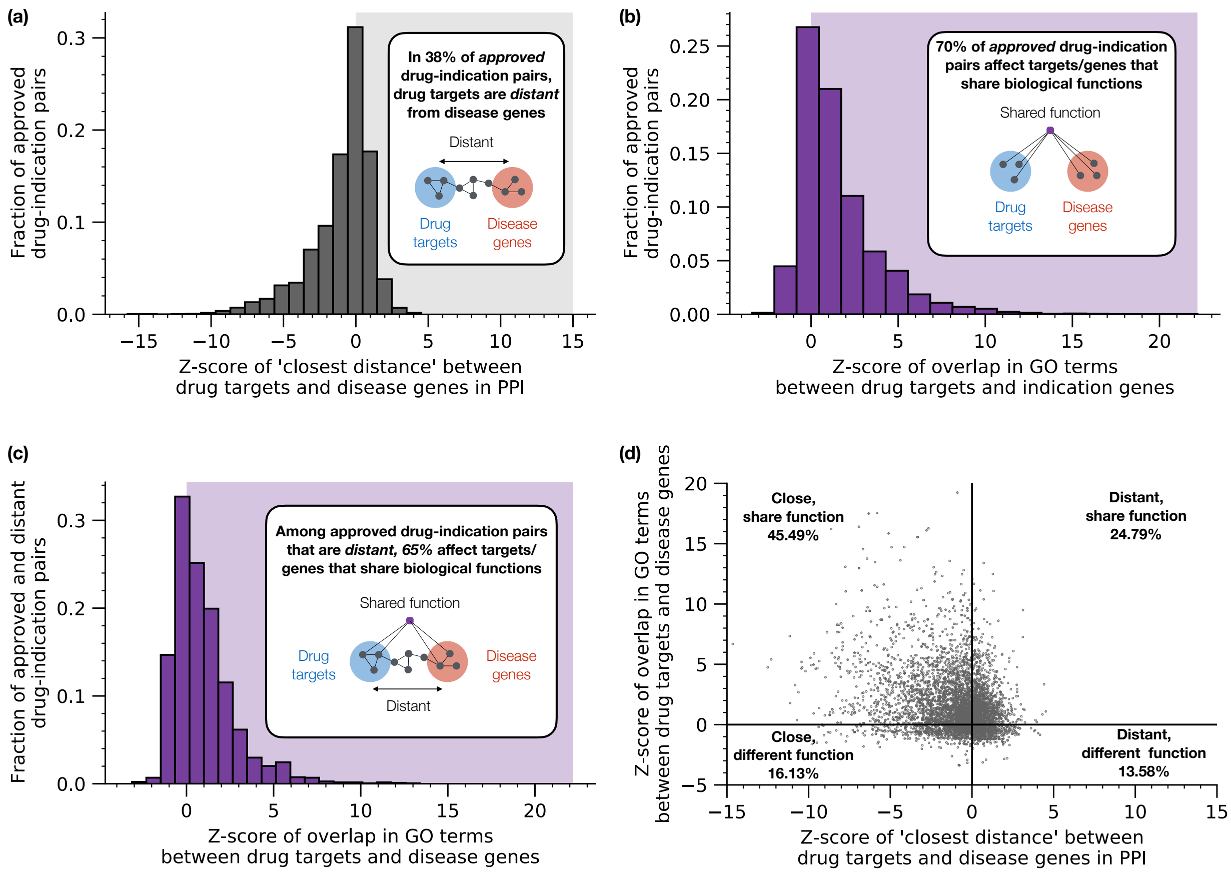

Supplementary Figure 3: Drugs may treat diseases by affecting the same functions, even when drug targets are far from disease genes. **(a)** In 38% of approved drug-indication pairs, drug targets are farther from disease genes than is expected by random chance. Distance between drug targets and disease genes is calculated according to a state-of-the-art proximity metric[1,2] (Methods). **(b)** In 70% of approved drug-indication pairs, drug targets affect the same biological functions as disease genes more often than is expected by random chance, suggesting that biological functions are broadly important in modeling treatment. The functional overlap metric used is the Z scored intersection of Gene Ontology (GO) term multisets (Methods). **(c)** Among distant drug-indication pairs, 65% affect the same biological functions more often than is expected by random chance, suggesting that biological functions offer a powerful and complementary approach to explaining drug treatment. **(d)** Drugs treat diseases by either targeting proteins close to the disease genes (16.13% of approved drug-indication pairs), targeting proteins that affect the same biological functions as the disease genes (24.79%), or both (45.49%). Molecular-scale interactome approaches can only model proximity-based treatments. By integrating both physical interactions and biological functions, the multiscale interactome can model treatments that harness physical proximity, functional overlap, or both.

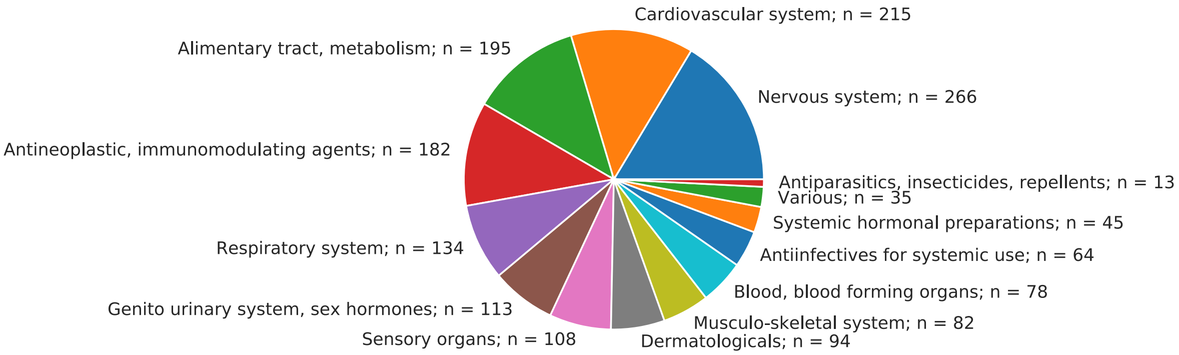

Supplementary Figure 4: The multiscale interactome models treatments across human anatomy. The multiscale interactome includes approved drug-disease treatments across human anatomy as represented by the Anatomical Therapeutic Chemical Classification (ATC). Each drug is mapped to its ATC Level I class. Numbers represent the number of unique drugs per class.

Method	AUROC	Avg Prec	Rec@50
Jaccard Similarity of GSEA GO Term Sets	0.500	0.024	0.219
Intersection of GSEA GO Term Sets	0.500	0.022	0.217
Jaccard Similarity of GO Term Sets	0.564	0.048	0.235
Intersection of GO Term Sets	0.558	0.045	0.213
Jaccard Similarity of GO Term Multisets	0.560	0.041	0.216
Intersection of GO Term Multisets	0.554	0.040	0.208
Z Scored Jaccard Similarity of GO Term Sets	0.533	0.039	0.211
Z Scored Intersection of GO Term Sets	0.551	0.040	0.207
Z Scored Jaccard Similarity of GO Term Multisets	0.533	0.035	0.205
Z Scored Intersection of GO Term Multisets	0.553	0.039	0.196
Max of Resnik Similarity	0.557	0.026	0.229
Average of Resnik Similarity	0.558	0.035	0.219
Best Match Average of Resnik Similarity	0.528	0.049	0.222
Max of SimIC	0.555	0.029	0.228
Average of SimIC	**0.573**	0.035	0.235
Best Match Average of SimIC	0.554	0.043	0.219
SimGIC	0.558	**0.050**	**0.237**
**Multiscale Interactome**	**0.705**	**0.091**	**0.347**
**Multiscale Interactome vs Best GO Baseline**	**+23.0%**	**+82.0%**	**+46.4%**

Supplementary Figure 5: The multiscale interactome significantly outperforms baseline methods that incorporate functional information when predicting what drugs treat a disease. The multiscale interactome substantially outperforms all Gene Ontology (GO) baseline methods in predicting what drugs will treat a given disease. Compared to the best GO baseline method, the multiscale interactome improves AUROC by 23.0% (0.705 vs. 0.573), Average Precision by 82.0% (0.091 vs. 0.050), and Recall@50 by 46.4% (0.347 vs. 0.237) (described values in bold). GO baselines are described in Methods. Reported values are averaged across five-fold cross validation.

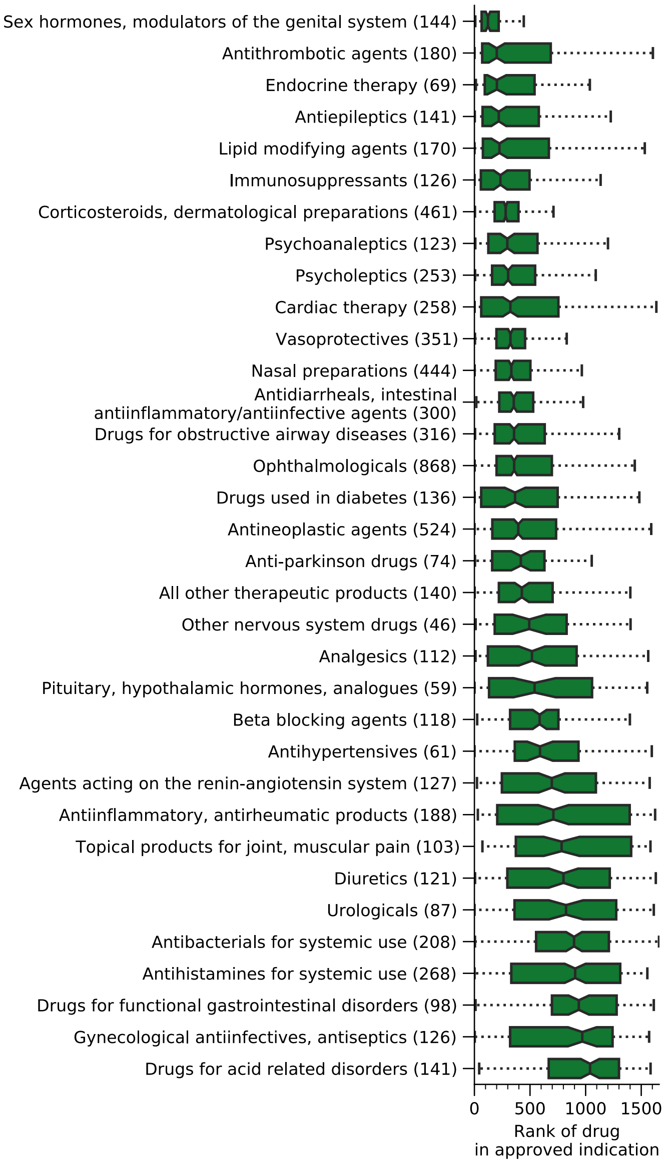

Supplementary Figure 6: Predictive power of multiscale interactome by drug category. The multiscale interactome predicts what drugs treat a given disease (Figure 2a-c, Methods). For each Anatomical Therapeutic Chemical Classification (ATC) Level II drug class, the rank of the drug in its approved indication is shown. Boxplots: median (line); 95% CI (notches); 1st, 3rd quartiles (boxes); data within $1.5\times$ the inter-quartile range from the 1st, 3rd quartiles (whiskers). Sample sizes in parentheses.

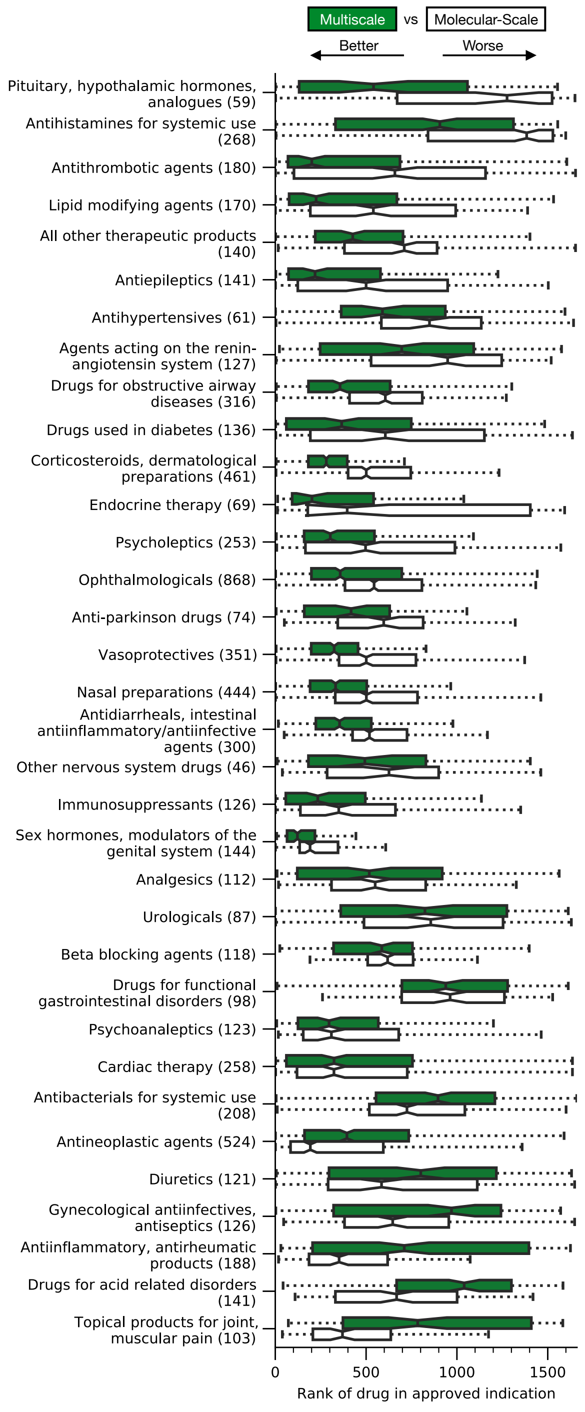

Supplementary Figure 7: Predictive power of multiscale interactome vs molecular-scale interactome by drug category. The multiscale interactome predicts what drug will treat a given disease more accurately than a molecular-scale interactome approach using diffusion profiles (Methods). For each Anatomical Therapeutic Chemical Classification (ATC) Level II drug class, the rank of the corresponding drugs in their approved indications is shown for both the multiscale interactome and the molecular-scale interactome. Drug classes are ordered by how much the multiscale interactome outperforms the molecular-scale interactome. Boxplots: median (line); 95% CI (notches); 1st, 3rd quartiles (boxes); data within $1.5\times$ the inter-quartile range from the 1st, 3rd quartiles (whiskers). Sample sizes in parentheses.

Method	AUROC	Avg Prec	Rec@50
Molecular-scale Interactome	**0.620**	**0.065**	**0.264**
Multiscale Interactome + Uberon	**0.715**	**0.091**	**0.343**
Multiscale Interactome + Cell Ontology	0.710	0.092	0.329
Multiscale Interactome + Uberon + Cell Ontology	0.702	0.090	0.335
Multiscale Interactome + Uberon vs. Molecular-scale interactome	**+15.3%**	**+40.0%**	**+29.9%**

Supplementary Figure 8: Alternative multiscale interactomes that explicitly model cells, tissues, organs, and the relationships between them also outperform molecular-scale interactomes on a drug-indication prediction task. The best alternative multiscale interactome (multiscale interactome + Uberon) outperforms a molecular-scale interactome by 15.3% in AUROC (0.715 vs. 0.620), 40.0% in Average Precision (0.091 vs. 0.065), and 29.9% in Recall@50 (0.343 vs. 0.264) (described values in bold). Uberon[22,23] and the Cell Ontology[24,25] are anatomical ontologies with explicit representations of cells, tissues, organs, and the relationships between them. We use human-specific subsets of both when constructing the alternative multiscale interactomes (Methods, Supplementary Note 4). Reported values are averaged across five-fold cross validation.

Method	AUROC	Avg Prec	Rec@50
**Multiscale interactome without GO Filtering**	0.706	0.092	0.346
**Multiscale interactome with GO filtering**	0.705	0.091	0.347

Supplementary Figure 9: Filtering the Gene Ontology to only include biological functions associated with at least one drug target or disease protein does not substantially affect the performance of the multiscale interactome when predicting what drugs treat a disease. Details provided in Supplementary Note 5.

Method	AUROC	Avg Prec	Rec@50
Multiscale interactome without differentiating GO relation types	0.705	0.091	0.347
Multiscale interactome with differentiating GO relation types	0.709	0.088	0.335

Supplementary Figure 10: Augmenting the multiscale interactome so it differentiates relation types in the Gene Ontology does not significantly improve performance on a drug-indication prediction task. Details provided in Supplementary Note 5.

Method	AUROC	Avg Prec	Rec@50
**Multiscale interactome without differentiating disease-protein associations types**	0.705	0.091	0.347
**Multiscale interactome with differentiating disease-protein associations types**	0.708	0.099	0.337

Supplementary Figure 11: Augmenting the multiscale interactome so it differentiates between disease-protein associations that are based on effects like mutation, post-translational modification, or gene expression does not substantially affect the performance of the multiscale interactome when predicting what drugs treat a disease. Details provided in Supplementary Note 5.

Drug-Drug Comparison Method	Spearman ρ	p-value
**GO Term Overlap**	0.144	0.077
**GSEA Term Overlap**	0.179	0.028
**Diffusion Profile Similarity**	**0.392**	**$5.8x10^{-7}$**

Supplementary Figure 12: Diffusion profiles provide a more biologically relevant metric for drug-drug similarity than alternative functional approaches. We measure the Spearman correlation between the similarity of two drugs according to their gene expression signatures and the similarity of two drugs according to three drug-drug comparison methods: (1) intersection in Gene Ontology (GO) terms associated with drug targets and disease proteins, (2) intersection in GO terms enriched among drug targets and disease proteins according to Gene Set Enrichment Analysis[3], and (3) similarity in diffusion profiles (Methods). The similarity of two drugs according to the similarity of their diffusion profiles provides the strongest and most significant correlation with the similarity of the same drugs according to their gene expression signatures (Spearman $\rho = 0.392$; $p = 5.8x10^{-7}$, two-sided, $n = 152$; Figure 2f; in bold).

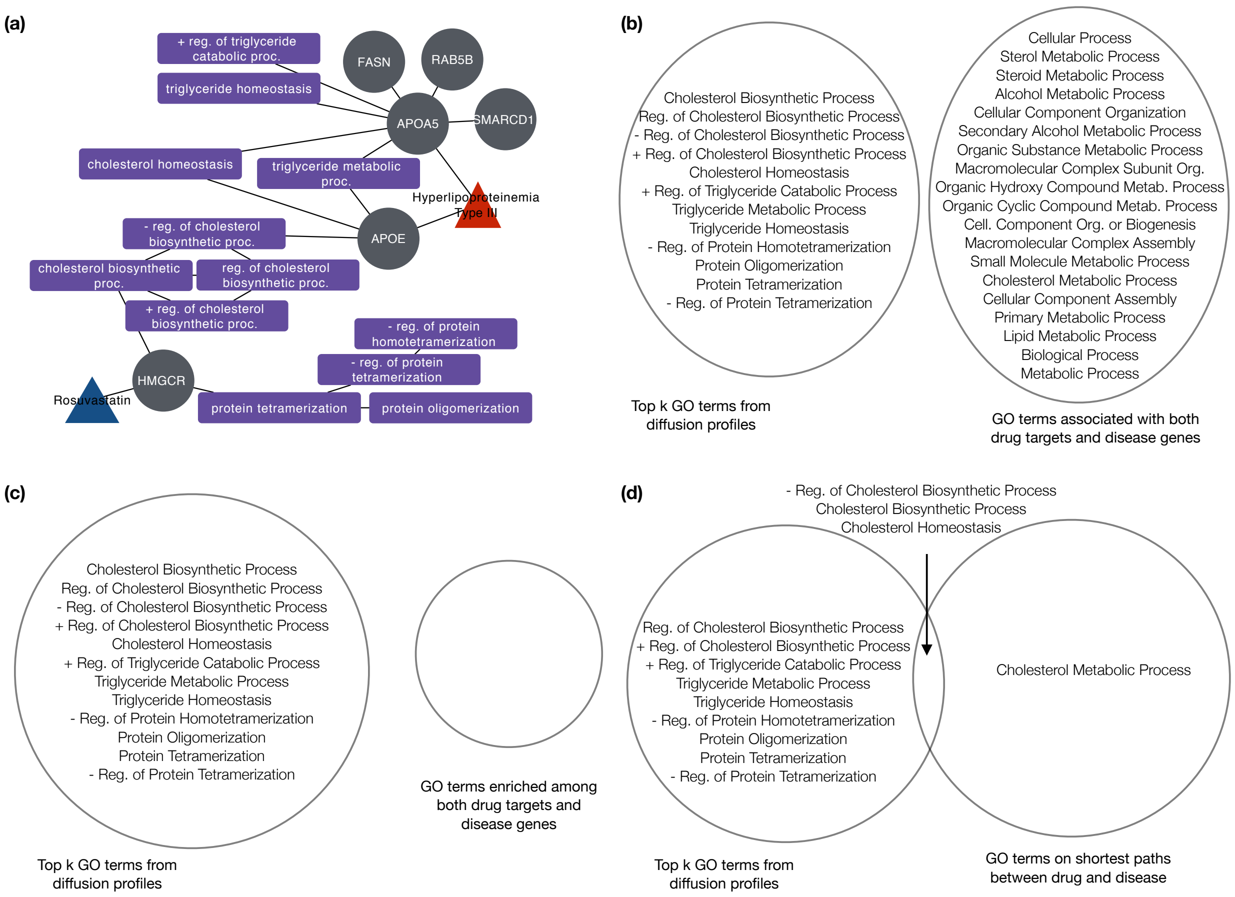

Supplementary Figure 13: Diffusion profiles identify the biological functions used by Rosuvastatin to treat Hyperlipoproteinemia Type III more effectively than alternative approaches. **(a)** The induced subgraph of proteins and biological functions identified via diffusion profiles as relevant to the treatment of Hyperlipoproteinemia Type III by Rosuvastatin. **(b, c, d)** Venn Diagrams compare the biological functions identified via diffusion profiles to the biological functions identified via **(b)** Gene Ontology (GO) terms associated with both drug targets and disease genes, **(c)** GO terms enriched among both drug targets and disease genes according to Gene Set Enrichment Analysis[3], and **(d)** GO terms on shortest paths between drug targets and disease genes. Diffusion profiles identify the key biological functions involved in Rosuvastatin's treatment of Hyperlipoproteinemia Type III more effectively than alternative approaches (Supplementary Note 3)[11–17]. Abbreviations: reg., regulation; org., organization; metab., metabolic; cell., cellular; +, positive; -, negative.

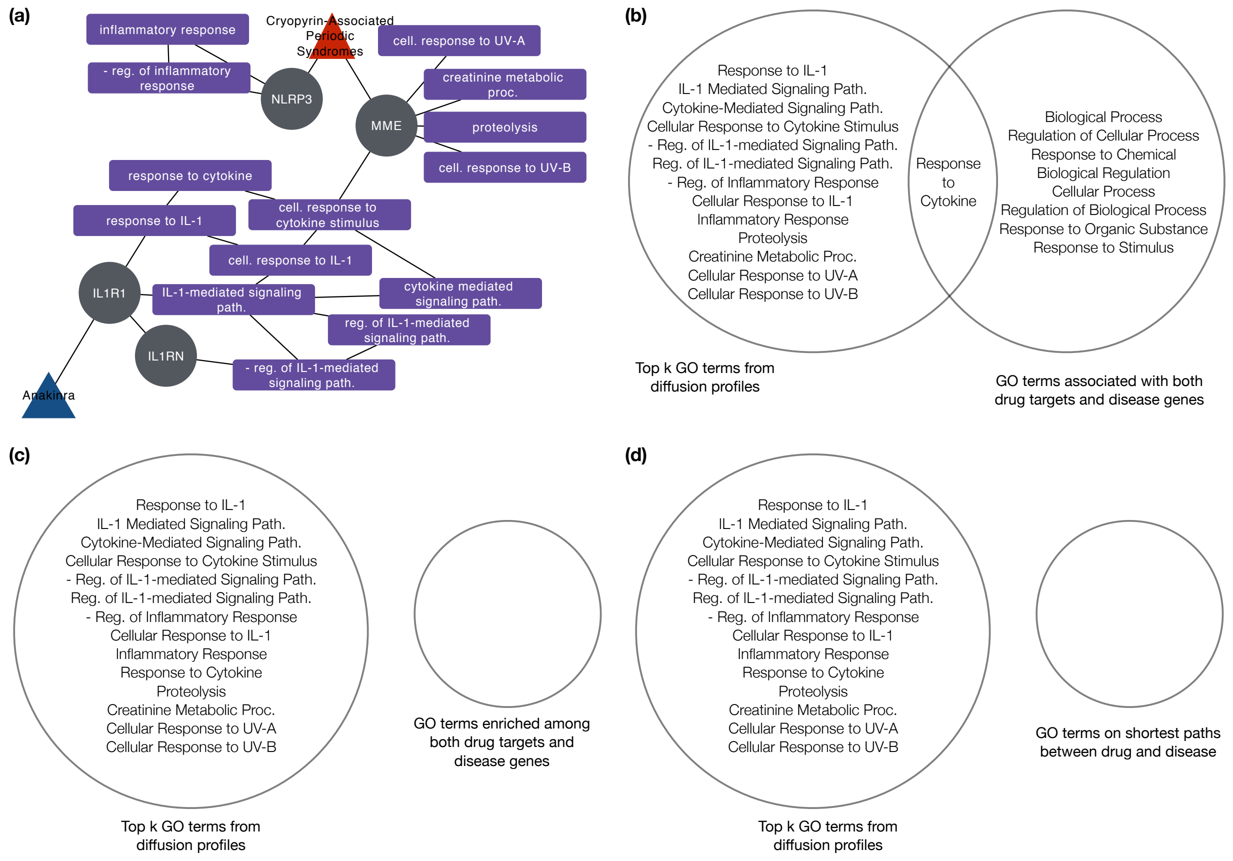

Supplementary Figure 14: Diffusion profiles identify the biological functions used by Anakinra to treat Cryopyrin-Associated Periodic Syndromes more effectively than alternative approaches. **(a)** The induced subgraph of proteins and biological functions identified via diffusion profiles as relevant to the treatment of Cryopyrin-Associated Periodic Syndromes by Anakinra. **(b, c, d)** Venn Diagrams compare the biological functions identified via diffusion profiles to the biological functions identified via **(b)** Gene Ontology (GO) terms associated with both drug targets and disease genes, **(c)** GO terms enriched among both drug targets and disease genes according to Gene Set Enrichment Analysis[3], and **(d)** GO terms on shortest paths between drug targets and disease genes. Diffusion profiles identify the key functions involved in Anakinra's treatment of Cryopyrin-Associated Periodic Syndromes—regulation of immune-mediated inflammation through the Interleukin-I beta signaling pathway[8–10]—but alternative approaches do not (Supplementary Note 3). Abbreviations: reg., regulation; path., pathway; proc., process; cell., cellular; +, positive; -, negative.

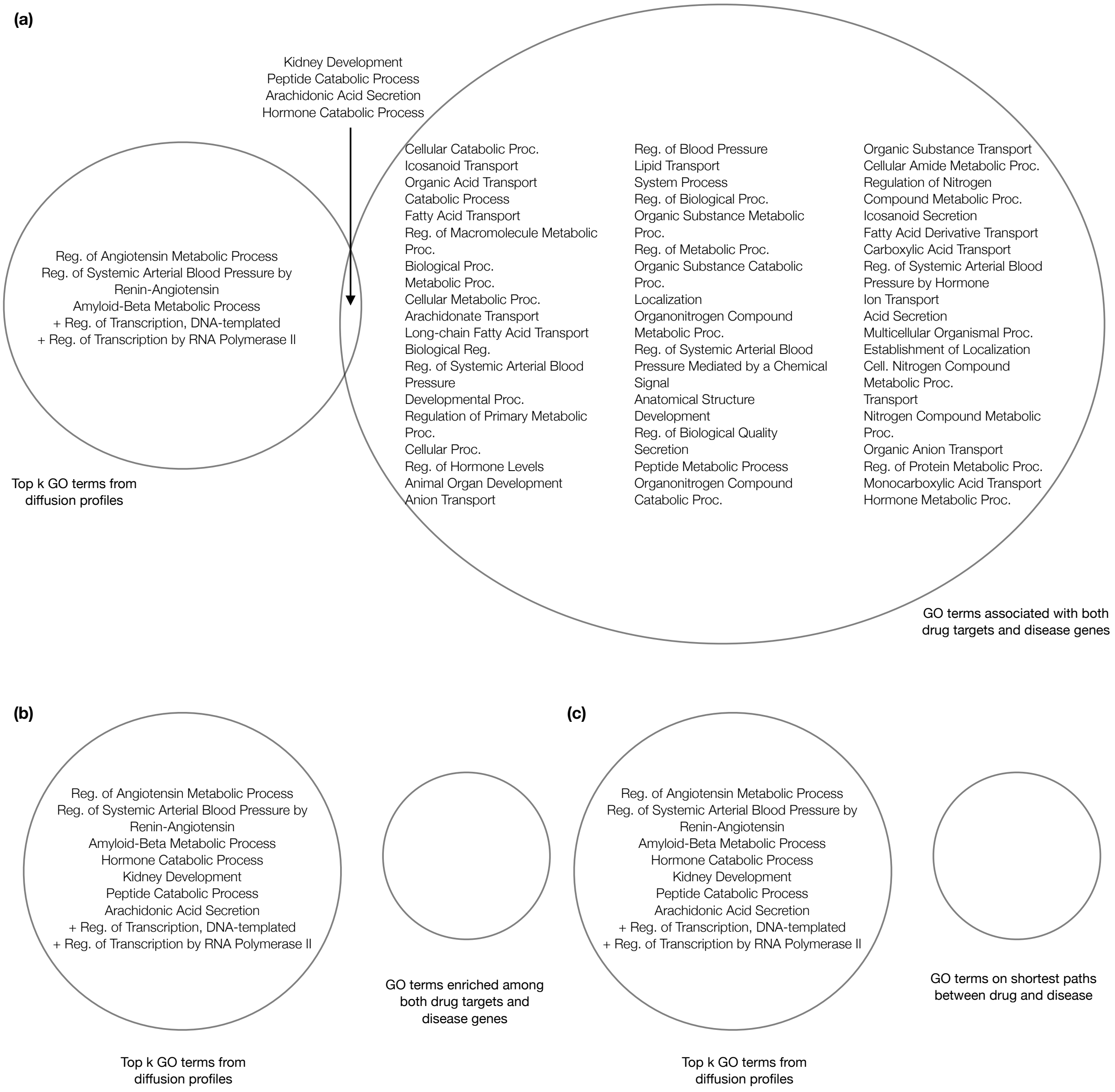

Supplementary Figure 15: Diffusion profiles identify the biological functions used by Benazepril to treat Hypertensive Disease more effectively than alternative approaches. (a, b, c) Venn Diagrams compare the biological functions identified as relevant to the treatment of Hypertensive Disease by Benazepril via diffusion profiles to the biological functions identified via **(a)** Gene Ontology (GO) terms associated with both drug targets and disease genes, **(b)** GO terms enriched among both drug targets and disease genes according to Gene Set Enrichment Analysis[3], and **(c)** GO terms on shortest paths between drug targets and disease genes. Diffusion profiles uniquely identify the renin-angiotensin pathway as a key biological function involved in treatment, helping explain why mutations in Angiotensinogen (*AGT*) alter the efficacy of treatment[?,26–29]. Abbreviations: reg., regulation; proc., process; cell., cellular; +, positive; -, negative.

	Rosuvastatin	Hyperlipoproteinemia Type III
1	HMGCR	APOE
2	Cholesterol Biosynthetic Process	APOA5
3	Protein Tetramerization	RAB5B
4	Protein Oligomerization	SMARCD1
5	Negative Regulation of Cholesterol Biosynthetic Process	FASN
6	Positive Regulation of Cholesterol Biosynthetic Process	Triglyceride Homeostasis
7	Regulation of Cholesterol Biosynthetic Process	Cholesterol Homeostasis
8	Negative Regulation of Protein Homotetramerization	Positive Regulation of Triglyceride Catabolic Process
9	Negative Regulation of Protein Tetramerization	Triglyceride Metabolic Process
10	Protein Heterotetramerization	Acylglycerol Homeostasis
11	Protein Homotetramerization	Triglyceride Catabolic Process
12	Regulation of Protein Tetramerization	Positive Regulation of Lipoprotein Lipase Activity
13	FGF1	Lipid Transport
14	Sterol Biosynthetic Process	Positive Regulation of Fatty Acid Biosynthetic Process
15	Cholesterol Metabolic Process	Positive Regulation of Lipid Catabolic Process
16	MVK	Positive Regulation of Very-Low-Density Lipoprotein Particle Remodeling
17	ERLIN2	Osteoblast Diifferentiation
18	Secondary Alcohol Biosynthetic Process	Antigen Processing and Presentation
19	HMGCL	Chromatin Remodeling
20	PMVK	Nucleosome Disassembly

Supplementary Figure 16: Top 20 proteins and biological functions for the treatment of Hyperlipoproteinemia Type III by Rosuvastatin. Proteins and biological functions are ranked according to their visitation frequency in the drug and disease diffusion profiles (Methods).

	Anakinra	Cryopyrin Associated Periodic Syndromes
1	IL1R1	MME
2	Interleukin-1 Mediated Signaling Pathway	NLRP3
3	Response to Interleukin-1	Proteolysis
4	Cellular Response to Interleukin-1	Cellular Response to UV-B
5	Negative Regulation of Interleukin-1-Mediated Signaling Pathway	Cellular Response to UV-A
6	Regulation of Interleukin-1-Mediated Signaling Pathway	Cellular Response to Cytokine Stimulus
7	Response to Cytokine	Creatinine Metabolic Process
8	Cytokine-Mediated Signaling Pathway	Negative Regulation of Inflammatory Response
9	IL1RN	Inflammatory Response
10	IRAK1	Positive Regulation of Interleukin-1 Beta Secretion
11	IRAK2	Positive Regulation of NF-kappaB Transcription Factor Activity
12	ZNF675	Negative Regulation of NF-kappaB Transcription Factor Activity
13	VRK2	Negative Regulation of Acute Inflammatory Response
14	TRAF6	Positive Regulation of Cysteine-Type Endopeptidase Activity Involved in Apoptotic Process
15	IRAK3	Negative Regulation of Interleukin-1 Beta Secretion
16	MYD88	Cellular Response to Lipopolysaccharide
17	PRKCA	Negative Regulation of NF-kappaB Import Into Nucleus
18	SRC	Cellular Response to UV
19	Cellular Response to Cytokine Stimulus	Negative Regulation of Acute Inflammatory Response to Antigenic Stimulus
20	Positive Regulation of Transcription by RNA Polymerase II	Cytoplasmic Sequestering of NF-kappaB

Supplementary Figure 17: Top 20 proteins and biological functions for the treatment of Cryopyrin Associated Periodic Syndromes by Anakinra. Proteins and biological functions are ranked according to their visitation frequency in the drug and disease diffusion profiles (Methods).

	Benazepril	Diltiazem	Hypertensive Disease
1	ACE	KCNA5	Positive Regulation of Transcription by RNA Polymerase II
2	Arachidonic Acid Secretion	CACNA1C	Cilium Assembly
3	Peptide Catabolic Process	CACNA2D1	Positive Regulation of Transcription, DNA-templated
4	Regulation of Systemic Arterial Blood Pressure by Renin-Angiotensin	CACNA1S	EGFR
5	Kidney Development	HTR3A	Negative Regulation of Transcription by RNA Polymerase II
6	Amyloid-Beta Metabolic Process	CACNG1	ATP1A1
7	Hormone Catabolic Process	LMNA	FN1
8	Regulation of Angiotensin Metabolic Process	VAPA	TP53
9	BDKRB2	Calcium Ion Transport	LZTFL1
10	COMT	Muscle Contraction	TGFB1
11	AGXT	Regulation of Heart Rate by Cardiac Conduction	Negative Regulation of Transcription, DNA-templated
12	ACTB	Cardiac Muscle Cell Action Potential Involved in Contraction	APOE
13	LMNA	Negative Regulation of Cell Proliferation	CUL3
14	EGR1	Regulation of Calcium Ion Transport	PRKACA
15	AGR2	Positive Regulation of Cell Aging	WNK4
16	MYH9	SRI	SMAD3
17	EWSR1	Regulation of Calcium Ion Transmembrane Transport via High Voltage-Gated Calcium Channel	TNF
18	CSNK2A2	Atrial Cardiac Muscle Cell Action Potential	IDUA
19	AGTR2	Potassium Ion Transmembrane Transport	CAV1
20	CSNK2A1	Regulation of Ventricular Cardiac Muscle Cell Membrane Repolarization	EDN1

Supplementary Figure 18: Top 20 proteins and biological functions for the treatment of Hypertensive Disease by Benazepril or Diltiazem. Proteins and biological functions are ranked according to their visitation frequency in the drug and disease diffusion profiles (Methods).

Supplementary references

1. Guney, E., Menche, J., Vidal, M. & Barábasi, A.-L. Network-based in silico drug efficacy screening. *Nature Communications* **7**, 10331 (2016).

2. Cheng, F. *et al.* Network-based approach to prediction and population-based validation of in silico drug repurposing. *Nature Communications* **9**, 2691 (2018).

3. Subramanian, A. *et al.* Gene set enrichment analysis: a knowledge-based approach for interpreting genome-wide expression profiles. *Proceedings of the National Academy of Sciences* **102**, 15545–15550 (2005).

4. Barabási, A.-L., Gulbahce, N. & Loscalzo, J. Network medicine: a network-based approach to human disease. *Nature Reviews Genetics* **12**, 56–68 (2011).

5. Cowen, L., Ideker, T., Raphael, B. J. & Sharan, R. Network propagation: a universal amplifier of genetic associations. *Nature Reviews Genetics* **18**, 551–562 (2017).

6. Lotfi Shahreza, M., Ghadiri, N., Mousavi, S. R., Varshosaz, J. & Green, J. R. A review of network-based approaches to drug repositioning. *Briefings in Bioinformatics* **19**, 878–892 (2018).

7. Pushpakom, S. *et al.* Drug repurposing: progress, challenges and recommendations. *Nature Reviews Drug Discovery* **18**, 41–58 (2019).

8. Shinkai, K., McCalmont, T. & Leslie, K. Cryopyrin-associated periodic syndromes and autoinflammation. *Clinical and Experimental Dermatology: Clinical Dermatology* **33**, 1–9 (2008).

9. Kone-Paut, I. & Galeotti, C. Anakinra for cryopyrin-associated periodic syndrome. *Expert Review of Clinical Immunology* **10**, 7–18 (2014).

10. Wishart, D. S. *et al.* DrugBank 5.0: a major update to the DrugBank database for 2018. *Nucleic Acids Research* **46**, D1074–D1082 (2017).

11. Utermann, G., Jaeschke, M. & Menzel, J. Familial hyperlipoproteinemia type III: Deficiency of a specific apolipoprotein (APO E-III) in the very-low-density lipoproteins. *FEBS Letters* **56**, 352–355 (1975).

12. Utermann, G. *et al.* Polymorphism of apolipoprotein E: Genetics of hyperlipoproteinemia type III. *Clinical Genetics* **15**, 37–62 (1979).

13. Ghiselli, G., Schaefer, E. J., Gascon, P. & Breser, H. Type III hyperlipoproteinemia associated with apolipoprotein E deficiency. *Science* **214**, 1239–1241 (1981).

14. Wang, J. *et al.* APOA5 genetic variants are markers for classic hyperlipoproteinemia phenotypes and hypertriglyceridemia. *Nature Clinical Practice Cardiovascular Medicine* **5**, 730–737 (2008).

15. Evans, D., Seedorf, U. & Beil, F. Polymorphisms in the apolipoprotein a5 (APOA5) gene and type III hyperlipidemia. *Clinical Genetics* **68**, 369–372 (2005).

16. Moghadasian, M. H. Clinical pharmacology of 3-hydroxy-3-methylglutaryl coenzyme a reductase inhibitors. *Life Sciences* **65**, 1329–1337 (1999).

17. Holdgate, G., Ward, W. & McTaggart, F. Molecular mechanism for inhibition of 3-hydroxy-3-methylglutaryl CoA (HMG-CoA) reductase by rosuvastatin. *Biochemical Society Transactions* **31**, 528–531 (2003).

18. Consortium, G. O. The Gene Ontology in 2010: extensions and refinements. *Nucleic Acids Research* **38**, D331–D335 (2010).

19. Consortium, G. O. Gene Ontology Consortium: going forward. *Nucleic Acids Research* **43**, D1049–D1056 (2015).

20. Consortium, G. O. The Gene Ontology resource: 20 years and still GOing strong. *Nucleic Acids Research* **47**, D330–D338 (2018).

21. Ashburner, M. *et al.* Gene Ontology: tool for the unification of biology. *Nature Genetics* **25**, 25–29 (2000).

22. Mungall, C. J., Torniai, C., Gkoutos, G. V., Lewis, S. E. & Haendel, M. A. Uberon, an integrative multi-species anatomy ontology. *Genome Biology* **13**, R5 (2012).

23. Haendel, M. A. *et al.* Unification of multi-species vertebrate anatomy ontologies for comparative biology in Uberon. *Journal of Biomedical Semantics* **5**, 21 (2014).

24. Bard, J., Rhee, S. Y. & Ashburner, M. An ontology for cell types. *Genome Biology* **6**, R21 (2005).

25. Diehl, A. D. *et al.* The Cell Ontology 2016: enhanced content, modularization, and ontology interoperability. *Journal of Biomedical Semantics* **7**, 1–10 (2016).

26. Balfour, J. A. & Goa, K. L. Benazepril. *Drugs* **42**, 511–539 (1991).

27. Lavoie, J. L. & Sigmund, C. D. Minireview: overview of the renin-angiotensin system—an endocrine and paracrine system. *Endocrinology* **144**, 2179–2183 (2003).

28. Caulfield, M. *et al.* Linkage of the angiotensinogen gene to essential hypertension. *New England Journal of Medicine* **330**, 1629–1633 (1994).

29. Jeunemaitre, X. *et al.* Molecular basis of human hypertension: role of angiotensinogen. *Cell* **71**, 169–180 (1992).
