# Supplementary material for: Identification of disease treatment mechanisms through the multiscale interactome
Source: Nat Commun. 2021 Mar 19;12:1796. doi: 10.1038/s41467-021-21770-8 (PMC7979814; doi:10.1038/s41467-021-21770-8)
Supplement: Supplementary file 2 — Description of Additional Supplementary Files [file 41467_2021_21770_MOESM2_ESM.pdf]

Description of Additional Supplementary Files

Code and additional instructions for using data can be found at https://github.com/snap-stanford/multiscale-interactome.

File Name: Supplementary Data 1
Description: Supplementary dataset of interactions between drugs and proteins.
Columns and data: Columns include node_1, node_2, node_1_type, node_2_type, node_1_name, and node_2_name. Entries in node_1 and node_2 correspond to IDs for the given node: DrugBank IDs are used for drugs, Entrez IDs are used for proteins, UMLS CUI IDs are used for indications, GO IDs are used for biological functions. Columns referring to node types denote the type of node (drug, protein, indication, or biological_function).

File Name: Supplementary Data 2
Description: Supplementary dataset of interactions between diseases and proteins.
Columns and data: Columns include node_1, node_2, node_1_type, node_2_type, node_1_name, and node_2_name. Entries in node_1 and node_2 correspond to IDs for the given node: DrugBank IDs are used for drugs, Entrez IDs are used for proteins, UMLS CUI IDs are used for indications, GO IDs are used for biological functions. Columns referring to node types denote the type of node (drug, protein, indication, or biological_function).

File Name: Supplementary Data 3
Description: Supplementary dataset of interactions between proteins and proteins.
Columns and data: Columns include node_1, node_2, node_1_type, node_2_type, node_1_name, and node_2_name. Entries in node_1 and node_2 correspond to IDs for the given node: DrugBank IDs are used for drugs, Entrez IDs are used for proteins, UMLS CUI IDs are used for indications, GO IDs are used for biological functions. Columns referring to node types denote the type of node (drug, protein, indication, or biological_function).

File Name: Supplementary Data 4
Description: Supplementary dataset of interactions between proteins and biological functions.
Columns and data: Columns include node_1, node_2, node_1_type, node_2_type, node_1_name, and node_2_name. Entries in node_1 and node_2 correspond to IDs for the given node: DrugBank IDs are used for drugs, Entrez IDs are used for proteins, UMLS CUI IDs are used for indications, GO IDs are used for biological functions. Columns referring to node types denote the type of node (drug, protein, indication, or biological_function).

File Name: Supplementary Data 5
Description: Supplementary dataset of hierarchy of interactions between biological functions
Columns and data: Columns include node_1, node_2, node_1_type, node_2_type, node_1_name, and node_2_name. Entries in node_1 and node_2 correspond to IDs for the given node: DrugBank IDs are used for drugs, Entrez IDs are used for proteins, UMLS CUI IDs are used for indications, GO IDs are used for biological functions. Columns referring to node types denote the type of node (drug, protein, indication, or biological_function).

File Name: Supplementary Data 6
Description: Supplementary dataset of approved drug-disease pairs
Columns and data: IDs correspond to DrugBank IDs for drugs and UMLS CUI IDs for indications.

File Name: Supplementary Data 7

Description: Supplementary dataset of drug classes according to Anatomical Therapeutic Chemical Classification
Columns and data: Columns include db_id (the DrugBank ID for the drug), atc_code (the full ATC code for the drug), and then multiple levels of the ATC code mapped by the code corresponding to that level and the corresponding name.

File Name: Supplementary Data 8
Description: Supplementary dataset of selected gene expression signatures from the Broad Connectivity Map
Columns and data: The first six columns describe metadata for each gene expression signature: the drug column describes the DrugBank ID of the drug, the drug_name column is the drug's name, the sig_id column is the signature ID assigned by the Broad Connectivity Map, and the cell, dose, and time columns describe the conditions under which the signature was generated by the Broad Connectivity Map. The remaining columns are Entrez IDs for genes and the corresponding L5 signatures measured and computed by the Broad Connectivity Map.

File Name: Supplementary Data 9
Description: Supplementary dataset of genetic mutations that alter drug response from PharmGKB
Columns and data: DrugBank IDs are used for drugs, UMLS CUI IDs are used for diseases, Entrez IDs are used for genes. Clinical Annotation IDs are IDs provided by PharmGKB for the corresponding treatment alteration.

File Name: Supplementary Data 10
Description: Supplementary dataset of optimized diffusion profiles
Columns and data: This folder contains the optimized diffusion profiles used in the main paper. Each file is a numpy (".npy") array. The beginning of the file name corresponds to either the DrugBank ID or name for the corresponding drug or the UMLS CUI ID or name for the corresponding disease. Each entry in the numpy array corresponds to the visitation frequency of a given node in the corresponding diffusion profile. To map each index of the numpy array to the corresponding node or vice-versa, the files "idx2node.pkl" and "node2idx.pkl" can be used. Finally, a reference graph "di_protein_go_graph.pkl" is provided here. This graph is principally provided to verify the accurate construction of the multiscale interactome from the manuscript.
