# Supplementary material for: Identification of disease treatment mechanisms through the multiscale interactome
Source: Nat Commun. 2021 Mar 19;12:1796. doi: 10.1038/s41467-021-21770-8 (PMC7979814; doi:10.1038/s41467-021-21770-8)
Supplement: Supplementary file 13 — Reporting Summary [file 41467_2021_21770_MOESM13_ESM.pdf]

nature research

Corresponding author(s): Jure Leskovec

Last updated by author(s): Jan 27, 2021

Reporting Summary

Nature Research wishes to improve the reproducibility of the work that we publish. This form provides structure for consistency and transparency in reporting. For further information on Nature Research policies, see our Editorial Policies and the Editorial Policy Checklist.

Statistics

For all statistical analyses, confirm that the following items are present in the figure legend, table legend, main text, or Methods section.

n/a	Confirmed	
☐	☒	The exact sample size (*n*) for each experimental group/condition, given as a discrete number and unit of measurement
☒	☐	A statement on whether measurements were taken from distinct samples or whether the same sample was measured repeatedly
☐	☒	The statistical test(s) used AND whether they are one- or two-sided<br>*Only common tests should be described solely by name; describe more complex techniques in the Methods section.*
☐	☒	A description of all covariates tested
☐	☒	A description of any assumptions or corrections, such as tests of normality and adjustment for multiple comparisons
☐	☒	A full description of the statistical parameters including central tendency (e.g. means) or other basic estimates (e.g. regression coefficient) AND variation (e.g. standard deviation) or associated estimates of uncertainty (e.g. confidence intervals)
☐	☒	For null hypothesis testing, the test statistic (e.g. *F*, *t*, *r*) with confidence intervals, effect sizes, degrees of freedom and *P* value noted<br>*Give P values as exact values whenever suitable.*
☒	☐	For Bayesian analysis, information on the choice of priors and Markov chain Monte Carlo settings
☒	☐	For hierarchical and complex designs, identification of the appropriate level for tests and full reporting of outcomes
☐	☒	Estimates of effect sizes (e.g. Cohen's *d*, Pearson's *r*), indicating how they were calculated

Our web collection on statistics for biologists contains articles on many of the points above.

Software and code

Policy information about availability of computer code

Data collection	All analyses were performed using Python 3.7, NetworkX 2.3, NumPy 1.16.2, Pandas 0.24.2, Scipy 1.3.0, GOATOOLS 0.8.4. Code and data are available at github.com/snap-stanford/multiscale-interactome. Additional packages used are in the requirements.txt file at the GitHub repository.
Data analysis	All analyses were performed using Python 3.7, NetworkX 2.3, NumPy 1.16.2, Pandas 0.24.2, Scipy 1.3.0, GOATOOLS 0.8.4. Code and data are available at github.com/snap-stanford/multiscale-interactome. Additional packages used are in the requirements.txt file at the GitHub repository.

For manuscripts utilizing custom algorithms or software that are central to the research but not yet described in published literature, software must be made available to editors and reviewers. We strongly encourage code deposition in a community repository (e.g. GitHub). See the Nature Research guidelines for submitting code & software for further information.

Data

Policy information about availability of data

All manuscripts must include a data availability statement. This statement should provide the following information, where applicable:

- Accession codes, unique identifiers, or web links for publicly available datasets
- A list of figures that have associated raw data
- A description of any restrictions on data availability

All data used in the paper, including the multiscale interactome, approved drug-disease treatments, drug and disease classifications, gene expression signatures, and pharmacogenomic relationships is publicly available at github.com/snap-stanford/multiscale-interactome. This manuscript uses and compiles data from numerous public data sources including: DrugBank (https://go.drugbank.com/), the Drug Repurposing Hub (https://clue.io/repurposing), the Drug Repurposing Database (http://apps.chiragjpgroup.org/repoDB/), the Drug Indication Database, DisGeNet (https://www.disgenet.org/), Disease Ontology (https://disease-

ontology.org/, HUGO (https://www.genenames.org/), the Unified Medical Language System (https://www.nlm.nih.gov/research/umls/index.html), the Biological General Repository for Interaction Datasets (https://thebiogrid.org/), the Database of Interacting Proteins (https://dip.doe-mbi.ucla.edu/dip/Main.cgi), the Human Reference Protein Interactome Mapping Project (http://www.interactome-atlas.org/), Menche-2015, the Gene Ontology and Gene Ontology Plus (http://geneontology.org/), the Broad Connectivity Map (https://clue.io/cmap), the Pharmacogenomics Knowledgebase (https://www.pharmgkb.org/), Uberon (http://uberon.github.io/), the Cell Ontology (http://www.obofoundry.org/ontology/cl.html), and the Human Cell Atlas Ontology (https://github.com/HumanCellAtlas/ontology).

Field-specific reporting

Please select the one below that is the best fit for your research. If you are not sure, read the appropriate sections before making your selection.

[x] Life sciences [ ] Behavioural & social sciences [ ] Ecological, evolutionary & environmental sciences

For a reference copy of the document with all sections, see nature.com/documents/nr-reporting-summary-flat.pdf

Life sciences study design

All studies must disclose on these points even when the disclosure is negative.

Sample size	Sample sizes for experiments and compiled data are provided in the figure legends and the Methods. We selected the databases used in this study in order to allow for the largest possible systematic study of drug-disease treatments that used high quality, experimentally-validated pharmacological relationships (detailed in Methods). The appropriateness of each specific database for its corresponding pharmacological relationship is detailed in Methods. The appropriateness of the total number of databases for the downstream analyses is exemplified through the results.
Data exclusions	When compiling data from the relevant databases, we followed guidelines from each database to ensure we used only high quality experimental data and avoided introducing circularity into our analyses (detailed in Methods).
Replication	No new experimental findings are reported in this manuscript. Reproducibility of the computational analyses in the manuscript are ensured through clear representation of the methods used and the public release of both code and data. The findings in this study are based on a random walk-based model with optimized hyperparameters as described in the manuscript. The predictive power of this model was replicated in numerous sweeps described in Supplementary Notes 2, 4, 5 and Supplementary Figures 8-11. All attempts at replication were successful and the resulting analyses are based on the model as described in the Methods.
Randomization	No samples/organisms/participants were used.
Blinding	Blinding is not relevant to the study as there was no group allocation involved. In cases where categorical analyses were conducted (i.e. analysis across drug classes), the categories were determined externally and not by the investigators (i.e. the drug classes used here are from the Anatomical Therapeutic Classification). The findings in this study are based on a random walk-based model described in the manuscript and the resulting analyses are based on this model.

Reporting for specific materials, systems and methods

We require information from authors about some types of materials, experimental systems and methods used in many studies. Here, indicate whether each material, system or method listed is relevant to your study. If you are not sure if a list item applies to your research, read the appropriate section before selecting a response.

Materials & experimental systems

n/a	Involved in the study
[x]	[ ] Antibodies
[x]	[ ] Eukaryotic cell lines
[x]	[ ] Palaeontology and archaeology
[x]	[ ] Animals and other organisms
[x]	[ ] Human research participants
[x]	[ ] Clinical data
[x]	[ ] Dual use research of concern

Methods

n/a	Involved in the study
[x]	[ ] ChIP-seq
[x]	[ ] Flow cytometry
[x]	[ ] MRI-based neuroimaging
